# Risk Factors for Sudden Cardiac Death or Unexplained Death in Patients Treated with Clozapine: A Systematic Review

**DOI:** 10.1192/bjo.2026.11234

**Published:** 2026-06-30

**Authors:** Tejas Easwar, Damodar Chari, Shahbaz Abdullah, Hugh Tollinton, Elizabeta Mukaetova-Ladinska

**Affiliations:** 1 University of Cambridge, Cambridge, United Kingdom; 2 Leicestershire Partnership NHS Trust, Old Age Psychiatry, Leicester, United Kingdom; 3 Leicestershire Partnership NHS Trust, Psychiatry, Leicester, United Kingdom; 4 University of Leicester, Leicester, United Kingdom

## Abstract

**Aims:**

Clozapine is the gold-standard treatment for treatment-resistant schizophrenia but carries serious cardiac risks, including sudden cardiac death (SCD). Specific risk factors for SCD in clozapine-treated patients remain poorly defined.

Aims were to systematically identify and synthesise evidence on risk factors for SCD and sudden unexplained death (SUD) in clozapine-treated patients, to guide clinical monitoring.

**Methods:**

We conducted a systematic review following PRISMA guidelines (PROSPERO: CRD420250646384). Five databases were searched from inception to 13 October 2025. Studies reporting SCD or SUD in clozapine-treated patients were included without restrictions on study design, demographics, or diagnosis. Two reviewers independently screened studies and extracted data. Quality was assessed using JBI checklists and ROBINS-E tools. Given study heterogeneity, we performed structured narrative synthesis.

**Results:**

Twenty-one studies (1989–2023) were included, comprising 498 cases of SCD/SUD in clozapine-treated patients. Risk factors were grouped into four categories: treatment intensity (high doses 525 mg/day, rapid titration), drug interactions (valproate, benzodiazepines, polypharmacy), lifestyle factors (smoking, obesity, diabetes, substance use), and monitoring. Two patterns emerged: early inflammatory myocarditis (weeks 2–6) and late-onset cardiomyopathy (months–years).

**Conclusion:**

Clozapine-associated SCD appears multifactorial. These findings suggest a role for gradual titration, avoidance of high-risk co-medications, baseline biomarker monitoring, and ongoing management of metabolic and cardiovascular risk factors. Increased multidisciplinary surveillance may help identify patients at higher risk and inform clinical decision-making in clozapine-treated patients.

